# Editorial Comment: Sacral neuromodulation for neurogenic Lower Urinary Tract Dysfunction

**DOI:** 10.1590/S1677-5538.IBJU.2023.02.02

**Published:** 2023-05-29

**Authors:** Marcio Augusto Averbeck

**Affiliations:** 1 Hospital Moinhos de Vento Porto Alegre RS Brasil Chefe de Neuro-Urologia, Unidade de Videourodinâmica, Hospital Moinhos de Vento. Porto Alegre, RS, Brasil

Liechti MD ^1^, van der Lely S ^1^, Knüpfer SC ^1, 2^, Abt D ^3, 4^, Kiss B ^5^, Leitner L, ^1^, et al. *NEJM Evid 2022; 1 (11). Online ahead of print*

## COMMENT

This is a sham-controlled, double-blind, multicenter trial, which included patients with refractory neurogenic lower urinary tract dysfunction (NLUTD) at four Swiss referral centers (1). Patients underwent sacral neuromodulation (SNM) test phase with lead placement into the sacral foramina S3 (rarely, S4). Neurostimulator was implanted for permanent stimulation only in patients presenting ≥50% improvement in key bladder diary variables (successful test phase). For 2 months, neuromodulation was optimized using subsensory stimulation with individually adjusted parameters. Thereafter, the neurostimulator remained on or was switched off (1:1 random allocation to group SNM ON or SNM OFF, respectively) for 2 months, followed by a neurourologic reevaluation. The primary outcome was success, as defined above, of SNM compared with baseline.

Of 124 patients undergoing SNM test phase, 65 (52%) were classified as therapy responders. Of these, 60 patients were randomly assigned to the intervention. After 2 months of intervention, the SNM ON group demonstrated a success rate of 76%. In the SNM OFF group, 42% of patients showed sustained SNM effects despite their neurostimulator being switched off during the last 2 months (odds ratio, 4.35; 95% confidence interval, 1.43 to 13.21; P=0.009).

This the first well-designed RCT demonstrating that SNM effectively corrected refractory NLUTD in the short term in well-selected neuro-urological patients. The use of subsensory stimulation allowed switching off the implantable neurostimulator in the control group without jeopardizing blinding. Additionally, this study did not detect notable carryover effects (>2months), therefore supporting a need for continuous stimulation in neuro-urological patients. The heterogeneity of neurologic patient population, which precluded a disease-specific analysis, may be seen as the main limitation of this trial.
